# Terpenoids and Phytocannabinoids Co-Produced in *Cannabis Sativa* Strains Show Specific Interaction for Cell Cytotoxic Activity

**DOI:** 10.3390/molecules24173031

**Published:** 2019-08-21

**Authors:** Dvora Namdar, Hillary Voet, Vinayaka Ajjampura, Stalin Nadarajan, Einav Mayzlish-Gati, Moran Mazuz, Nurit Shalev, Hinanit Koltai

**Affiliations:** 1 Institute of Plant Sciences, Agricultural Research Organization, Volcani Center, Bet Dagan 7505101, Israel; 2 Israeli Gene Bank, Volcani Center, Bet Dagan 7505101, Israel

**Keywords:** *Cannabis sativa*, phytocannabinoids, terpenoids, entourage effect, cancer cell line

## Abstract

Mixtures of different *Cannabis sativa* phytocannabinoids are more active biologically than single phytocannabinoids. However, cannabis terpenoids as potential instigators of phytocannabinoid activity have not yet been explored in detail. Terpenoid groups were statistically co-related to certain cannabis strains rich in Δ^9^-tetrahydrocannabinolic acid (THCA) or cannabidiolic acid (CBDA), and their ability to enhance the activity of decarboxylase phytocannabinoids (i.e., THC or CBD) was determined. Analytical HPLC and GC/MS were used to identify and quantify the secondary metabolites in 17 strains of *C. sativa*, and correlations between cannabinoids and terpenoids in each strain were determined. Column separation was used to separate and collect the compounds, and cell viability assay was used to assess biological activity. We found that in “high THC” or “high CBD” strains, phytocannabinoids are produced alongside certain sets of terpenoids. Only co-related terpenoids enhanced the cytotoxic activity of phytocannabinoids on MDA-MB-231 and HCT-116 cell lines. This was found to be most effective in natural ratios found in extracts of cannabis inflorescence. The correlation in a particular strain between THCA or CBDA and a certain set of terpenoids, and the partial specificity in interaction may have influenced the cultivation of cannabis and may have implications for therapeutic treatments.

## 1. Introduction

Today, specific cannabis (*Cannabis sativa* L.) strains used in medical treatments are generally defined by the strain’s popular name and/or by its content of two phytocannabinoids: Δ^9^-tetrahydrocannabinol (THC) and cannabidiol (CBD). Phytocannabinoids are a structurally homogenous class of monoterpenoids indicative of *Cannabis sativa* L. [1]. THC and CBD are decarboxylated products of Δ^9^-tetrahydrocannabinolic acid (THCA) and cannabidiolic acid (CBDA), respectively, both produced by the plant [1]. Δ^9^-THC, its isomer Δ^8^-THC, and, to a lesser extent, their degradation derivative cannabinol (CBN), have been found to bind to the endocannabinoid receptors CB_1_ and CB_2_ [2].

Small and Beckstead [3] were the first to suggest a systematic chemotaxonomy of the hundreds of different hybrid cannabis strains. Active strains containing very high amounts of THC (>85% of the total phytocannabinoids extracted, with CBD < 0.5%) were classified as chemotype I; intermediate strains dominated by CBD but containing relative high amounts of THC were classified as chemotype II; and strains high in CBD (CBD > 85% of the total phytocannabinoids extracted) were classified as chemotype III [4,5].

However, this dichotomic (THC/CBD-based) chemotaxonomy ignores the fact that cannabis strains produce more than 600 different secondary metabolites, many of which are biologically active [6]. Among the various strains, tens of other phytocannabinoids (20 in average) and scores of terpenoids (i.e., various terpenes, terpenoids, and terpene alcohols) are produced by the plant [7,8]. Therefore, there are numerous and varied phytocannabinoid and terpenoid assemblages produced by different strains [9]. 

These compound assemblages are significant for cannabis therapies, since whole extracts of cannabis inflorescence, containing a plethora of chemical compounds, have been found to be more active than single, purified phytocannabinoids [10,11,12,13]. Among others, whole inflorescence extracts have been identified to alleviate chronic pain in humans [14] and animals [15], enhance cytotoxic activity against cancerous cell lines [12,13], and reduce seizures in both epileptic mice [16] and humans [17]. This phenomenon has been called “the entourage effect” [10] and may originate from synergistic interactions between cannabis compounds. In some cases of synergistic activity, the effect may originate from the activation of more than one signaling pathway [13,18].

Studies of the entourage effect in the last decade actually address two different entourage effects: the “intra-entourage effect” and “inter-entourage effect”. The former can be seen when the enhanced biological activity is attributed to the interactions between different phytocannabinoids present in a given strain. As de Meijer and Hammond [19] noted, cannabis plants never produce only one phytocannabinoid [20]. 

In contrast, the inter-entourage effect suggests that enhanced biological activity may be attributed to secondary metabolites—mainly terpenoids—produced by cannabis strains. Terpenoids are known for their medicinal properties including anti-inflammatory and anticancer activities [21,22,23], but here, in the general gist of the inter-entourage effect, they are considered as promoters and instigators of therapeutic phytocannabinoid activity. Although comprising only a few percent of the total secondary metabolites in cannabis flowers, the effect of terpenoids may be of great significance [10,21]. However, to date no study has directly investigated the phytocannabinoid–terpenoid inter-effect for separate families of compounds, nor explored the significance of the effect of terpenoids on the main phytocannabinoid activity according to a given strain.

In this paper we explore the effects of the terpenoid assemblage produced by different cannabis chemotypes on the biological activity of their respective phytocannabinoids.

## 2. Results

### 2.1. Phytocannabinoids–Terpenoids Multivariate Analysis

**Figure 1:**
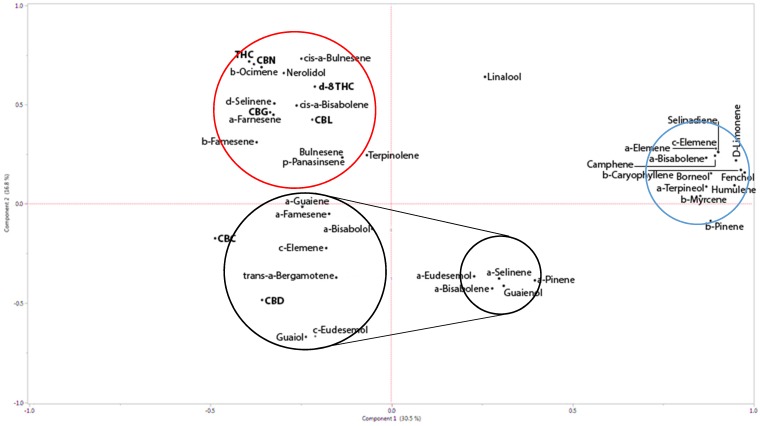
Principal Components Analysis (PCA) with phytocannabinoid–phytocannabinoid and phytocannabinoid–terpenoid correlations calculated for THCA and CBDA affinity. (For information regarding specific strains and chemotyping, see Supplementary Table S1). THCA co-related compounds are circled in red, while CBDA co-related compounds are circled in black. Strong correlations are indicated by larger circles, smaller correlations with smaller circles. Independent compounds, showing weak correlations to either THCA or CBDA are circled in blue. THCA: Δ^9^-tetrahydrocannabinolic acid; CBDA: cannabidiolic acid.

To identify the relationship between the primary phytocannabinoid and the terpenoids in the extracts of various strains, principle component analysis (PCA) was performed (see Supplementary Table S1). PCA indicated that the first two principal components explained most of the variation (47.3%; Figure 1). The first principal component had negative correlation with CBDA (r = −0.4), and the second principal component was positively co-related with THCA (r = 0.83) (Figure 1 and Supplementary Table S2). The PCA biplot identified three main groups, largely discriminated by their correlation to either CBDA, THCA, or independent terpenoids. In terms of the main compounds, only guaiol and eudesmol derivatives showed strong positive correlation with CBDA (Figure 1 and Supplementary Figure S1). 

The first and second components showed a reliable dependency of THCA and α-ocimene_mt_, cis-α-bulnesene_st_ and nerolidol_st_. CBDA showed strong dependence on particular sesquiterpenoids, namely guiaol_stol_, γ-eudesmol_stol_, trans-α-bergamotene_st_, γ-elemene_st_, α-bisabolol_stol,_ and α-farnesene_st_. Cannabigerolic acid (CBGA) was related to δ-selinene_st_, cis-α-bisabolene_st,_ and α-famesene_st_ (subscript abbreviations: mt—monoterpene; st—sesquiterpenes; stol—sesquiterpenes; dt—diterpene). Altogether, cannabichromenic acid (CBCA) demonstrated no correlation with any of the terpenoids in the extracted assemblage (Figure 1). Interestingly, although ocimene and guaiol are both guaienes, a closely related natural sesquiterpenoid with the molecular formula C_15_H_24_, ocimene was found to be statistically related with THC, while guaiene is related to CBD in cannabis inflorescences. 

PCA also demonstrated correlations between different phytocannabinoids. Despite the fact that CBGA is the precursor to the three dominant phytocannabinoids CBDA, THCA, and CBCA, a relatively stronger correlation was found between CBGA and THCA over both CBDA and CBCA (Figure 1). Similarly, although CBN is considered the natural decomposition byproduct of the three main phytocannabinoids (i.e., CBDA, THCA, and CBCA), the strongest correlation between two phytocannabinoids was between THC and CBN (0.88 positive, Figure 1 and Supplementary Figure S1). 

### 2.2. Phytocannabinoids–Terpenoids Column Separation

According to the PCA correlations above, we wished to separate terpenoids and phytocannabinoids. As di-terpenoids have similar affinities and polarities, and as terpenoids were not observable in routine preparative HPLC, the separation of the two molecular groups was not trivial. We developed an ad-hoc column separation method based on the polarity gradient (see the Materials and Methods section), which proved to be reproducible and quantifiable. Using the column separation method, phytocannabinoids and terpenoids were collected separately from two strains—Cs12, which is a high-THC strain, and Arbel, which is a high-CBD strain (Table 1). Moreover, we demonstrate that using this method we can separate terpenoids and phytocannabinoids into smaller, diverse compound assemblages, with different compositions and ratios between them, depending on polarity-based retention order and their column affinity (Table 1). 

Relative and absolute amounts of the collected fractions were calculated, as detailed in the Materials and Methods section. These calculations showed that terpenoids make up around 8–10% of the total phytocannabinoid fraction extracted by ethanol (Table 2A). Hexane extraction resulted in a relatively higher amount of terpenoids and alkanes, about 20% of the total extract (Table 2B).

### 2.3. Phytocannabinoids–Terpenoids Combinatory Cytotoxic Activity

The systematic correlation between a certain set of terpenoids and THCA or CBDA raises the question whether phytocannabinoid activity on human cells might be affected by the presence of the related terpenoids. A further question we wished to study was whether this correlation is functionally specific, i.e., do terpenoids related to CBDA enhance the biological activity of THC on human cells and vice versa. Since previous research suggested that phytocannabinoid cytotoxic activity is related to their decarboxylated forms [24,25], for this part of the study we used decarboxylated phytocannabinoids, collected from two strains—the THCA-rich Cs12 and the high-CBDA strain named Arbel (Table 1). From those two strains, the whole extract was column-separated and terpenoids assemblages which were previously recognized by us as THCA- or CBDA-related terpenes (Figure 1) were isolated and used (Fraction 6 for THCA-related terpenoids and Fraction 3 for CBDA-related terpenoids; See Table 1).

**Figure 2:**
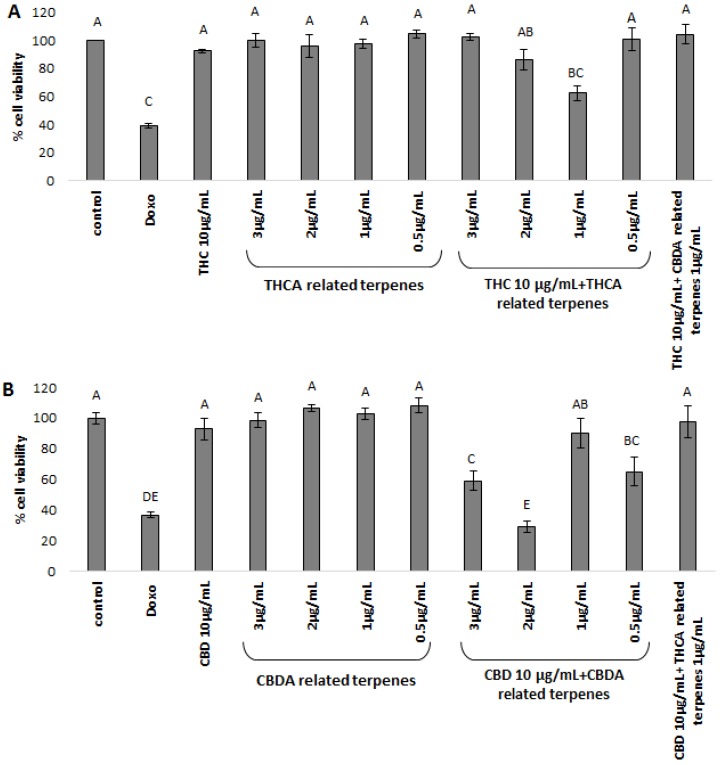
XTT cell viability assay of MDA-MB-231 cell line, treated for 48 h with (**A**) THC and terpene fraction; (**B**) CBD and terpene fraction. THC/CBD—in all treatments the phytocannabinoid concentration was 10µg/mL. The various concentrations of co-related terpenes and cross-related terpenes are detailed in the figure. Control—mixture of methanol and hexane (1:1), the solvent used for extraction and treatment. Doxo—doxorubicin 1 µg/µL. THCA-related terpenes—terpenes listed in Table 1A for Cs12 strain, fraction 6. CBDA-related terpenes—terpenes listed in Table 1B for the Arbel strain, fraction 3. THC + CBDA-related terpenes and CBD + THCA-related terpenes treatments were carried out at a ratio of 1:10 (1 µg/mL:10 µg/mL terpenoid:phytocannabinoid). Total amounts were extrapolated from the dry weight of the extracts and the relative amounts of the different molecules identified, as calculated from the area under the peak, valued by GC-personalized integration parameters (see Table 2). Percent cell viability is presented as mean ± SD. Columns with different letters are significantly different from all combination pairs, according to the Tukey–Kramer honest significant difference (HSD, *P* ≤ 0.05).

**Figure 3:**
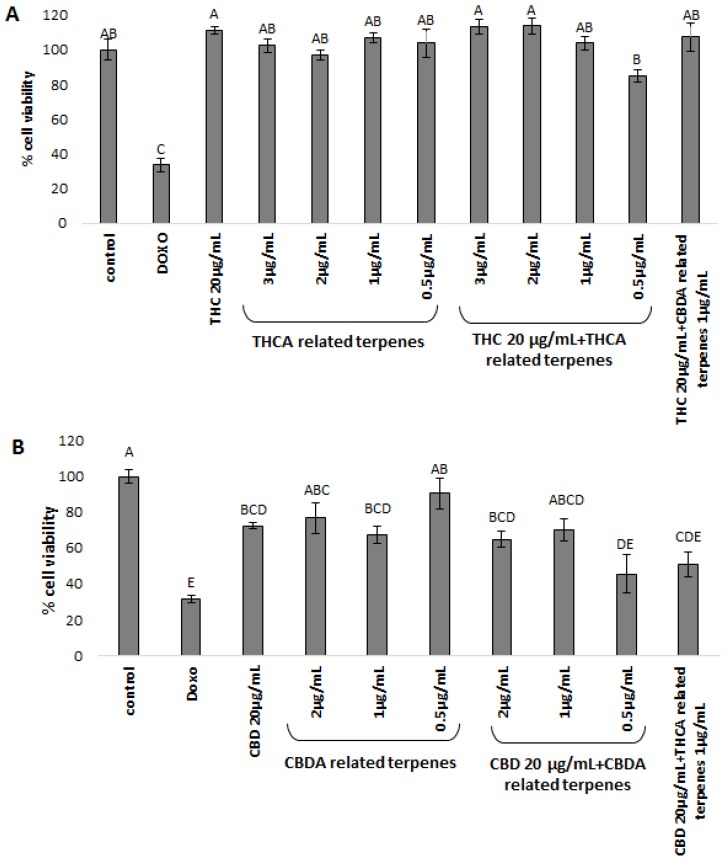
XTT cell viability assay of the HCT-116 cell line, treated for 48 h with (**A**) THC; and (**B**) CBD. THC/CBD—in all treatments the phytocannabinoid concentration was 20 µg/mL. The various concentrations of co-related terpenes and cross-related terpenes are detailed in the figure. Control—mixture of methanol and hexane (1:1), the solvent used for extraction and treatment. Doxo—doxorubicin 1 µg/µL. THCA-related terpenes—terpenes listed in Table 1A for Cs12 strain, fraction 6. CBDA-related terpenes—terpenes listed in Table 1B for the Arbel strain, fraction 3. THC + CBDA-related terpenes and CBD + THCA-related terpenes treatments were carried out at a ratio of 1:20 (1 µg/mL:20 µg/mL terpenoid:phytocannabinoid). Total amounts were extrapolated from the dry weight of the extract and the relative amounts of the different molecules identified, as calculated from the area under the peak, valued by GC-personalized integration parameters. Percent cell viability is presented as mean ± SD (uppercase letters mark statistical significance) of all treatments in replicas. Values with different letters are significantly different from all combinations of pairs, according to the Tukey–Kramer honest significant difference (HSD, *P* ≤ 0.05).

The separated subfractions were applied to two cancerous cell lines, MDA-MB-231 breast cancer and HCT-116 colorectal cancer cells, to investigate the biological implications and therapeutic effects of phytocannabinoid and terpenoid combinations. We used the collected subfractions alone and in specific combinations. The two cell lines were treated with purified, decarboxylated CBD or THC with or without the addition of statistically significant related terpenoids (see Figure 2 and Figure 3), at phytocannabinoid to terpenoid ratios observed in the whole inflorescence extract (Table 2). As detailed above, the terpenoid to phytocannabinoid ratio is approximately 1:10 for different cannabis strains grown under similar conditions (Table 2). To decide on the phytocannabinoid concentration to use for these assays, IC50 was determined for each of the cancer cell lines (Supplementary Figure S2). MDA-MB-231 was more sensitive to the cytotoxic activity of both THC and CBD than HCT-116 (Supplementary Figure S2). Therefore, treatments were set at 10 µg/mL and 20 µg/mL of each phytocannabinoid for MDA-MB-231 and HCT-116, respectively, combined with co-related terpenoids separated from the same strain in relative terpenoid to phytocannabinoid ratios of 0.25:10, 0.5:10, 1:10, 2:10, and 3:10. Finally, cross-related terpenoids, i.e., “CBDA-related terpenoids” with THC and “THCA-related terpenoids” with CBD (see Table 1 and Table 2), in terpenoids to phytocannabinoids ratios of 1:10 or 0.5:10 were also used for treatment.

MDA-MB-231 cell lines treated with 10 μg/mL THC alone showed only moderate cytotoxic activity, ~10% cell death (Figure 2A). THCA co-related terpenoids had no cytotoxic activity at the different concentrations examined. However, combinations mimicking natural variation, i.e., treatment with phytocannabinoids and their related terpenoids at naturally occurring terpenoid to phytocannabinoid ratios of 1:10, showed a significant increase in the levels of cell death for THCA-related terpenes coupled with THC (~40% cell death, Figure 2A). Increasing the amount of THCA-related terpenoids above natural ratios for treatments inhibited the biological activity of the combined compound to only ~15% cell death (Figure 2A). Cross-linking THC with CBDA-related terpenoids showed insignificant activity (Figure 2A). 

MDA-MB-231 cell lines treated with 10 μg/mL CBD showed minor cytotoxic activity, resulting in approximately ~7% cell death (Figure 2B). Treating the cell lines with the CBDA-related terpenoids alone showed no effect at the examined concentrations (Figure 2B). Combining CBD with CBDA-related terpenoids showed significant increases in the levels of cell death, to ~70% for terpenoid to phytocannabinoid ratios of 2:10 (Figure 2A). Similarly to THC and the THCA-related terpenoids, increasing the ratio of CBDA-related terpenoids in the treatment to 3:10 reduced cytotoxic activity (~40%, Figure 2B). Similarly to THC coupled with CBDA-related terpenoids, no significant cytotoxic activity was evident for CBD mixed with THCA-related terpenoids (Figure 2B). 

HCT-116 cell-lines treated with 20 μg/mL THC showed no cytotoxic activity, nor did the THCA co-related terpenoids alone (Figure 3A). However, the combination of THC and THCA-related terpenoids in the lowest amounts resulted in only limited cell death (~15% cell death, Figure 3A). THC mixed with CBDA-related terpenoids showed no activity (Figure 3A). 

HCT-116 cell-lines treated with 20 μg/mL CBD alone showed some cytotoxic activity, resulting in approximately ~30% cell death (Figure 3B). Treating the cells with the CBDA-related terpenoid mixture alone also showed some cytotoxic activity (~10–36%, Figure 3B). Combining CBD with CBDA-related terpenoids showed significant increases in cell death (~55%) at terpenoid to phytocannabinoid ratios of 0.5:20 (Figure 3B). In contrast to the MDA-MB-231 cell lines, CBD mixed with THCA-related terpenoids resulted in significant cytotoxic activity (~50% cell death, Figure 3B). 

These results point to the inter-entourage effect and, intriguingly, to terpenoid specificity. Terpenoid specificity was demonstrated to be more stringent for THC and have a higher degree of tolerance with CBD, at least for the HCT-116 cell line.

## 3. Materials and Methods

### 3.1. Materials

Seventeen different strains of *Cannabis sativa*, representing all three chemotypes, were analyzed using analytical high-pressure liquid chromatography (HPLC) (for cannabinoid profiling) and gas chromatography mass spectrometry (GC–MS) (for terpenoid identification and quantification, details on equipment below). This dataset was used for statistical analysis (see Methods section, below). Out of 17 strains, five (SCBD, Arbel, Paris, Roma, and DQ) are commercial strains purchased from Israeli Medical Cannabis (IMC, Israel). The other strains detailed in Supplementary Table S1 were grown in our greenhouse under conditions elaborated elsewhere [26]. Vegetative growth (20 h light/day for 32 days), followed by the flowering phase (12 h light/day for 35 days) was carried out under T5-36 W fluorescent tubes for vegetative growth, and under high-pressure sodium (HPS) 600 W bulbs tubes for flowering. Inflorescences were defined as “mature” once 70–80% of the pistils turned brown. An analytical balance (GR-120, A&D Company Ltd., Japan) was used to weigh each sample (e = 1 mg, d = 0.1 mg). 

As the main mode of action of phytocannabinoids is related with their decarboxylated form, (e.g., [27,28]), we heated the phytocannabinoids prior to biological treatment. For this, we collected pure phytocannabinoids from the whole inflorescence extract. First, a rough collection was done using preparative HPLC, and then the separated fraction was further purified by analytical HPLC coupled with the fraction collector. The accumulated pure phytocannabinoids (THCA from Cs12 and CBDA from Arbel) were freed of water in a lyophilizer and dried in a pressure tube under a stream of nitrogen. The dried pure phytocannabinoids were heated to 220 °C for 20 minutes to achieve full decarboxylation. 

### 3.2. Organic Solvents Extraction

Sixteen neutral and decarboxylated phytocannabinoid standards were utilized: cannabigerol (CBG & CBGA), cannabidiol (CBD & CBDA) and cannabidivarin (CBDV/A), Δ9-tetrahydrocannabinol (THC & THCA) and tetrahydrocannabivarin (THCV/A), cannabichromene (CBC), and cannabinol (CBN) and β-Caryophyllene and D-limonene (Restek, Bellefonte, PA, USA, purchased from Sigma-Aldrich, Rehovot, Israel., under Merck Millipore and LGC Standards). The standards were dissolved in methanol and used for quantification, retention time and elution order determination.

Organic solvents *n*-Hexane, ethanol, methanol, and ethyl acetate of high purity (EMSURE-grade for GC–MS; Mercury Scientific and Industrial Products Ltd.) were used in the extraction of cannabis inflorescences. Method blanks were routinely run with each extraction batch. Fresh *C. sativa* flowers (1–10 g each) were weighed and crushed by mortar and pestle after freezing with liquid nitrogen. The mortar and pestle were rinsed with acetone. The homogenized samples were placed in 15 mL tubes. To each sample, 4–40 mL of purified *n*-Hexane (ratio of 1:4, *w/v*) was added. The samples were shaken for 45 min at 200 rpm in a TU-400 orbital shaker incubator at room temperature. The supernatant containing the total extract from each sample was placed in a sterile 20 mL cantillation glass vial. No further treatment was carried out prior to separation on the silica column.

### 3.3. Column Separation

The separation method was developed using the *n*-hexanoic extract of two patented *C. sativa* L. strains, obtained from government-approved growers in Israel, Cs12 and Arbel. Cs12 is a chemotype I strain (see above), containing high amounts of THCA (>85% of total phytocannabinoids in the extract), while Arbel is a chemotype III strain, with high amounts of CBDA (>85% of total phytocannabinoids in the extract). Chemical analyses of the strains are detailed in Table 1. The separation experiment was repeated multiple times for verification.

Two milligrams of fresh inflorescence were extracted with 10 mL of *n*-hexane, EMSURE grade. The extract was dried under a gentle stream of nitrogen, and 300 µL of hexane was added to the sample. Eighteen grams of silica powder (230–400 mesh, 60 Å) was washed thoroughly with solvent and heated to 200 °C for 30 minutes. The clean silica powder was mixed with hexane to enable loading it on a 10 × 60 mm column. A thin layer (1 teaspoon) of alumina was placed on top of the silica. 

The whole extract of 300 µL (containing approximately 100 mg/mL) was loaded on the column top. The column was then washed with solvents of an increasing gradient of polarity, 20 mL per wash (Table 1). Three washes with hexane were followed by subsequent hexane:ethylacetate mixtures (at ratios 7:3, 1:1, 3:7, *v:v*), then flashed with 20 mL of ethyl acetate, and finally washed with ethanol. The fraction volume was reduced under a gentle stream of nitrogen, and 100 µL of each fraction was submitted to HPLC and GC–MS analyses. The separation was replicated six times, obtaining similar results. The solvent was dried under a very gentle stream of nitrogen without heating until completely dry, prior to biological tests.

Relative and absolute amounts of the collected fractions were calculated. Relative amounts were calculated based on peak area out of the total peak areas detected for the extract in GC–MS (for terpenoids and total decarboxylated phytocannabinoids, as phytocannabinoids go through spontaneous decarboxylation in the inlet) or analytical HPLC (for freshly extracted phytocannabinoids). Absolute amounts were calculated on the basis of the weight of the dried sub-fraction and against calibration curves of THCA and CBDA standards.

### 3.4. Compounds Identification by Gas Chromatography Coupled with Mass Selective Detection (GC–MS)

GC-MS analyses were carried out using an Agilent 7890B gas chromatograph coupled to a 5977A mass spectrometer (electron multiplier potential 2 kV, filament current 0.35 mA, electron energy 70 eV, and the spectra were recorded over the range of *m/z* 40 to 500). An Agilent 7683 autosampler was used for sample introduction. A 1 μL aliquot of each sample was injected into the GC–MS using a 1:10 split-ratio injection mode. Helium was used as the carrier gas at a constant flow of 1.1 mL s^−1^. An isothermal hold at 50 °C was maintained for 2 min, followed by a heating gradient of 6 °C min^−1^ to 300 °C, and the final temperature was held for 4 min. A 3 min solvent delay was applied. A 30 m, 0.25 mm ID, 5% cross-linked phenylmethyl siloxane capillary column (HP-5MS) with 0.25 μm film thickness was used for separation, and the injection port temperature was 220 °C. The MS interface temperature was 280 °C. 

Peak assignments were performed with a spectral library (NIST 14.0) and compared with MS data obtained from the injection of standards, purchased from LGC Standards (Teddington, UK). For identification and partial quantification, 10 µg of the aforementioned phytocannabinoid and terpenoid standards were injected to the GC–MS. 

### 3.5. Compound Identification by High-Pressure Liquid Chromatography (HPLC)

Carboxylated phytocannabinoids were detected, identified, and quantified using a Varian Prostar HPLC system coupled with a Varian 410 autosampler, 210 pump, and 320 UV/Vis detector. The separation was performed on a Purospher RP-18 end-capped column (250 mm × 4.6 mm ID; Merck KGaA, Darmstadt, Germany) with a guard column (4 mm × 4 mm ID). A 50 µL aliquot of sample was injected in isocratic separation mode, using 15% of 0.1% acetic acid in water and 85% methanol at a flow rate of 1.5 mL min^−1^ for 35 min. The compound peaks were detected for 220 nm and 280 nm. The main 16 phytocannabinoids (i.e., CBG, THC, CBD, CBC, and CBN) were identified using standards in both their carboxylated (for CBGA, THCA, CBDA) and neutral forms, trienyl and pentyl. Using the same method as detailed above for the samples, 5 ppm of the phytocannabinoid standards were dissolved in methanol and subjected to HPLC. THCA and THC were used as external calibration standards for the quantification of neutral and decarboxylated phytocannabinoids, respectively, at suitable concentrations between 5 and 40 µg. 

### 3.6. Determination of Extracts Cytotoxic Activity in Cell Lines Using XTT Viability Assay

Determination of the extract’s cytotoxic activity in cell lines using XTT Viability Assay were carried out on both MDA-MB-231 cell lines (ATCC HTB-26, mammary gland/breast adenocarcinoma derived from metastatic site) and HCT-116 (ATCC CCL-247, colorectal carcinoma). We chose these two cancerous cell lines as both were previously shown to be sensitive to the cytotoxic effect of cannabis, either to cannabinoids, crude extracts, or certain combinations of phytocannabinoids and terpenes (e.g., [12,13]). The relatively easy-to-quantify cell viability bioassay [29] used with two cannabis-sensitive cell lines makes it suitable as proof of concept vehicles.

The chosen cell lines, MDA-MB-231 and HCT-116 were grown at 37 °C in a humidified 5% CO_2_-95% air atmosphere. MDA-MB-231 cells were maintained in Dulbecco’s Modified Eagle’s medium (DMEM, Biological Industries, Beit Ha’emek, Israel). HCT-116 cells were maintained in McCOY’S 5A Modified medium (McCOY’S 5A, Biological Industries, Beit Ha’emek, Israel). Both media were supplemented with 10% fetal bovine calf serum (Biological industries, Beit Ha’emek, Israel), 1% of L-Glutamine, 1% of penicillin/streptomycin, and 0.02% Plasmocin (InvivoGen, San Diego, CA, USA).

Both cell lines (MDA-MB-231, HCT-116) were seeded into a 96 well plate at a concentration of 10,000 cells per well in triplicate in regular growing media. The following day, the media was replaced, and cells were treated with phytocannabinoids dissolved in regular growing media, doxorubicin (Sigma-Aldrich) as positive control (1 µg/mL) [30], 1% methanol + 0.1% hexane as negative control, and media only for blank. Different treatment concentrations of phytocannabinoids and/or terpenoids were added to separate wells. Cells were incubated for 48 h, after which an XTT reagent (2,3-Bis-(2-Methoxy-4-Nitro-5-Sulfophenyl)-2H-Tetrazolium-5-Carboxanilide) was used to quantify viability, according to the manufacturer’s instruction (Biological Industries, Beit Ha’emek, Israel). Cells were incubated with XTT reagent for 2 h at 37 °C in a humidified 5% CO_2_–95% air atmosphere. Absorbance was recorded by a SPEKTRA Fluor Plus photometer (Tecan, Salzburg, Austria) at 490 nm, with 650 nm of reference wavelength. Cell survival (% viability) was estimated using the equation: %cell survival = 100 × (A490 − A650) of treatment/(A490 − A650) of methanol + hexane control; A490 and A650 are the absorbencies of the XTT colorimetric reaction. Absorbance of medium alone (blank) was also deduced from specific readings. For determination of IC50, data points were connected by nonlinear regression lines of the sigmoidal dose-response relation. GraphPad Prism was used to produce dose-response curve and IC50 doses (Supplementary Figure S2).

### 3.7. Statistical Analysis and Correlation

Statistical analysis was carried out using the JMP statistical package and Microsoft Excel 2013 (for linear regression). Data are expressed as the mean ± standard deviation (SD) of n experiments. A minimum of three independent repetitions were conducted. Means of replicates were subjected to either Student’s t-test or the Tukey–Kramer honest significant difference (HSD; *P* ≤ 0.05), using the JMP statistical package, and considered significant when *P* ≤ 0.05.

The data on the 60 secondary metabolites detected in the 17 different varieties of cannabis were reduced by using Principal Component Analysis (PCA). In this way, the correlation between the secondary metabolites was utilized to create linear combinations of the data, independent of each other, preserving as much as possible the variation in the original measurements. These components (linear combinations) were extracted in descending order of importance, as expressed by the percentage of variance explained by each component. We extracted two such components, together explaining 47% of the variation in the original data. For each component, defined as a particular linear combination of the metabolite values, the coefficients (loadings) which were relatively large (in absolute value) highlight which metabolites were influential for that component.

For this purpose, full chemical analysis of the 17 *C. sativa* L. strains of both THC-rich and CBD-rich chemotypes were carried out using GC–MS combined with HPLC. The seventeen varieties analyzed are composed of four from our Greenhouse III, five from IMC, and eight from the gene bank collection (Israeli Gene Bank, Volcani Center, Bet Dagan, Israel). Examination of the pattern of loadings in Figure 1 shows which metabolites tend to cluster in the definition of each component.

## 4. Discussion

The aim of this research was to demonstrate the inter-entourage effect between phytocannabinoids and terpenoids and highlight potential applications in treatment. First, we showed significant correlation between certain phytocannabinoids and sets of terpenoids in a number of strains. These molecular correlations carry substantial implications. Terpenoids and cannabinoids bear no common precursors, nor similar biosynthetic pathways [28,29]. Thus, it is interesting to also consider how these chemical correlations developed in the evolutionary history of cannabis.

For examination of inter-entourage effect, we chose two cancerous cell lines that were previously shown to be sensitive to purified phytocannabinoid extracts in cell viability bioassays [12,13]. We revealed the inter-entourage activity in certain extracts, showing increased cytotoxic activity on cancerous cell lines by treatment with phytocannabinoids combined with low concentrations of co-related terpenoids. Moreover, mixing co-related terpenoids and phytocannabinoids (i.e., THCA-related terpenoids with THC or CBDA-related terpenoids with CBD) at ratios close to the natural plants showed the strongest effect. This increased activity may be the result of some preferential pathway in which the given terpenoids enhance the absorbance or activity of phytocannabinoids in the cells. More research should be done to characterize the related mode(s) of action.

Intriguingly, in contrast to the enhanced activity of THC coupled with THCA-related terpenoids, when THC was coupled with CBDA-related terpenoids no improvement in cytotoxic activity was observed. These results demonstrate specificity in terms of cytotoxic activity. Although less pronounced, similar results were obtained for CBDA, where mixtures of CBDA-related terpenoids with CBD dramatically increased cell mortality, while the addition of THCA-related terpenoids to CBD showed lower activity, especially for the HCT-116 cell-line. 

Two recent studies challenged the concept of the inter-entourage effect and concluded against its reliability. Blasco-Benito et al. [12] added a cocktail of several terpenoids, including β-pinene, linalool, β-caryophyllene, α-humulene, and nerolidol to THC at the same concentrations present in the inflorescence extract but did not record any enhancement in cytotoxic activity over that of THC by itself. It was suggested by the authors that other compounds (or compound combinations) present in the crude extract of cannabis are responsible for the superior potency over purified THC [12]. This accords with our results. Our correlation plot shows that while nerolidol has relative affinity to THCA, the other terpenoids described in their paper range from having no affinity to THCA to having minor affinity to CBDA (Figure 2 and Figure 3; see also [8,9]). This may explain the lack of activity observed when those specific terpenoids were added to THC. According to our results, THC activity is enhanced only by its co-related terpenoids, while other terpenoids inhibit its biological activity. Nevertheless, to fully decipher the biological mechanisms that underlie the inter-entourage effect, detailed characterization of the interaction between the specific phytocannabinoids and their co-related terpenoids is needed.

In another study of the inter-entourage effect, Santiago et al. [31] added monoterpenes—separately and as a mixture—of α-pinene and β-pinene, linalool, limonene and β-myrcene, and the sesquiterpene β-caryophyllene (30–100 µM each) to 10 µM of THC in order to examine the effect terpenoids may or may not have on CB1 and/or CB2 receptors. Our results fit the negative results Santiago et al. [31] present. As demonstrated in our results, using terpenoids such as the monoterpenes chosen in [31] that showed no statistical correlation to THCA, together with THC, in high concentrations of terpenoids not present in the plant, is not efficient. Our results strongly support their conclusion that “terpenoids are important for the behavioral effect of Cannabis”.

Theoretically, traditional cultivation, maintained through selective breeding over millennia, may have led to the creation of plants that bio-produce certain phytocannabinoids accompanied by specific sets of terpenes. Interest in the therapeutic uses of cannabis has a long history that continues until today. The earliest archaeological evidence for the use of cannabis is dated to 5000–6000 years ago in Central Asia and China [32]. Ancient Greek and Roman texts [33], as well as those of India and China [34] refer to specific medical uses for the plant. The preference to breed or select strains with particular combinations of phytocannabinoids and terpenoids may be associated with increased bio-activity on the human body. This hypothesis of intentional selective breeding is reassured by the biological efficacy we found for the phytocannabinoid–terpenoids treatments. It is possible that plants exhibiting these particular combinations of phytocannabinoids and terpenoids were chosen for cultivation due to their increased activity. Selective cultivation has been shown for other plant species, such as legumes [35]. Researchers studying plant evolution strongly argue that such developments cannot be a “fortuitous accumulation of a series of chance discoveries outside the faculty of man” [36]. Rather it is conscious human choice based on floral, environmental, and seasonal knowledge that resulted in plant domestication [37]. 

Our results may have also important therapeutic implications. As stated above, *C. sativa* is known to produce hundreds of metabolites, many of which have therapeutic potential. The possible permutations for different combinations of compounds, phytocannabinoids, and terpenoids in a given strain is a challenge for the development of cannabis-based drugs. The specificity in phytocannabinoid–terpenoid correlations and interaction may imply that during pharmaceutical development the inter-entourage effect of its components should be tested for antagonistic, additive, or synergistic results [10,11,18]. Controlling the ratios between terpenoids and phytocannabinoids in a given preparation may substantially improve biological activity, clarifying and improving potential therapies [10]. 

The given terpenoids examined (Supplementary Table S3) showed no activity on cancerous cell lines, as others also showed [12,31]. Therefore, terpenoids alone are not dose-dependent, and are utterly not active in the relevant dosages found in the plant. The effective cannabinoids–terpenoids combination also demonstrate a nonlinear dose dependency, rather the activity is dependent on a specific ratio—or ratio ranges—resembling that produced by the plant (Supplementary Table S3). Cannabinoids–terpenoids ratios significantly higher than those produced by the plant failed to activate the cell death mechanism [31]. This counter-intuitive dose specificity may be requisite to achieve the entourage effect.

Although we did not explore in this paper all possible phytocannabinoid and terpenoid combinations, it is worth noting that this project aimed to shed light on the concept rather than suggest any specific treatment. Moreover, we used mixtures of terpenes and terpenoids and did not investigate the activity potential for any specific terpene or terpenoid. Such research is beyond the scope of this paper. Instead, we highlight here the importance of a deeper characterization of secondary metabolites in cannabis and the impact that these secondary metabolites have on the activity of the major phytocannabinoids. The general enhanced biological activity exhibited on both cell lines indicates that phytocannabinoids coupled with co-related terpenoids show a clear inter-entourage effect and specificity. One conclusion that can be drawn from this demonstration is that the traditional “strain-centric” view of cannabis plants is dated and should shift to combinatorics design. Different combinations of terpenoids and phytocannabinoids may bear higher therapeutic potential. 

On the other hand, the differences in cell viability of the cancerous cell lines treated with similar ratios of phytocannabinoids and terpenoids stress the importance of an in-depth study of the active pharmaceutical ingredients (API) in relation to cannabis extracts. Deep profiling, followed by an investigation into biological potency, may be a novel approach more suitable for drug development than strain-centric methodology. By changing the focus of drug design from whole extracts to API composition, a mixture of compounds isolated from different strains may be used to achieve certain effective combinations and/or treatments [18]. 

Moreover, this study showed that cytotoxicity efficacy was significantly dependent on slight changes in the concentration of terpenoids in a given mixture. This may suggest that the degradation of terpenoids could also affect therapies. Thus, attention to terpenoid and cannabinoid degradation should be factored into Good Manufacturing Practices (GMP) to promote medical grade products. Finally, combinations of phytocannabinoid and terpenoid subfractions in the treatment of various medical conditions should be studied further to decipher both the inter-and intra-entourage effects. 

## 5. Conclusions

In this paper we present a clear example of the inter-entourage effect on cytotoxic activity. We showed: (1) A significant correlation between certain terpenoids and the main phytocannabinoids in various *C. sativa* strains. (2) Terpenoids, present in relatively minute amounts in cannabis extracts and possessing no therapeutic effect by themselves at these concentrations, add to the cytotoxicity of the dominant phytocannabinoid. This demonstrates the inter-entourage effect in cannabis treatments. (3) The inter-entourage interaction is specific, in part, since THC activity was enhanced only by its co-related terpenoids, at terpenoid to phytocannabinoid ratios found naturally in the plant, while CBD was more tolerant. (4) The relative ratio of phytocannabinoid to terpenoids, demonstrating the enhanced biological activity, entitled entourage effect, showed nonlinear dose dependency, rather a dose-specificity mode of action. 

It is possible that the significant correlation between certain terpenoids and phytocannabinoids is due to a long history of human cultivation, manipulated over many millennia of use. This proof of concept lays grounds for further research in formulating specific combinations with a desired therapeutic effect, and will impact the medical use and development of cannabis drugs.

## Figures and Tables

**Table 1:** Bulk separation results from the silica column, based on polarity and affinity of terpenes and phytocannabinoids extracted from two commercial strains of cannabis: Cs12 (chemotype I) and Arbel (chemotype III). Hex: *n*-hexane; EtAc: ethyl acetate; EtOH: ethanol; CBL: cannabicyclol; CBC: cannabichromene; CBD: cannabidiol; THC: tetrahydrocannabinol; CBG: cannabigerol.

Fraction No.	Solvent	Compounds in Fractions and Their Relative Abundances					
		A. Cs12				B. Arbel	
		Amount (mg)	Compound i.d.	%	Amount (mg)	Compound i.d.	%
1	Hex	0.3			2	alkanes	
2	Hex	0.1			18.4	α-Pinene Camphene β-Pinene alkanes	23 1 11 64
3	Hex	3.7	alkanes		6.2	α-Pinene β-Pinene D-Limonene trans-α-Bergamotene Aromadendrene γ-Selinene Guaiadiene δ-Selinene Selinadiene β-Maaliene	4 6 38 12 20 3 3 6 4 2
4	Hex:EtAc 7:3	11.1	alkanes		0.6		
5	Hex:EtAc 1:1	0.3	α-Pinene Camphene β-Pinene (+)3-Carene	36 2 43 19	47	Linalool Fenchol Terpinolene β-Famesene Humulne α-Famesene cis-α-Bergamotene Guaiol γ-Eudesmol α-Eudesmol α-Bisabolol phytol CBL CBC CBD δ8-THC δ9-THC CBG γ-Sitosterol	2 3 1 2 1 1 1 1 2 3 1 1 1 1 5 42 8 14 5
6	Hex:EtAc 3:7	73.2	β-Thujene α-Pinene Camphene β-Pinene α-Phellandrene α-Terpinene β-Phellandrene isosativene α-Guaiene	3 2 1 23 9 15 23 15 9	66	Linalool Fenchol Terpinolene Guaiol β-Selinene γ-Eudesmol α-Eudesmol phytol CBC CBD δ8-THC δ9-THC CBG	1 2 1 5 1 2 2 4 9 53 2 13 3
7	EtAc	3	β-Pinene α-Phellandrene α-Terpinene β-Phellandrene cis-α-Bergamotene isosativene trans-α-Bergamotene α-Guaiene α-Curcumene	1 8 1 18 3 3 23 43 1	22	γ-Eudesmol α-Eudesmol CBL CBC CBD δ8-THC δ9-THC CBG ketone	5 2 1 4 53 9 2 13 3
8	EtOH	57.6	β-Myrcene α-Phellandrene 2-Carene D-Limonene β-Ocimene γ-Terpinolene 3-Carene Citral m-Cymenol cis-α-Bergamotene Caryophyllene trans-α-Bergamotene α-Guaiene β-Famesene Humulne Giuaia-9,11-diene α-Bulnesene β-phellandrene α-Bisabolene Nerolidol α-Bisabolol phytol CBC CBD THC CBG	4 1 3 4 1 12 1 1 1 9 1 4 3 1 5 4 1 1 2 1 1 1 1 3 31 3	0.1	Linalool oxide Limonene oxide hydroxylinalool γ-Eudesmol α-Eudesmol alkanes CBL CBC CBD δ8-THC δ9-THC CBG	1 0.3 1 2 2 4 6 1 60 2 15 4

**Table 2:** Relative amounts of phytocannabinoids and terpenoids from different chemotype strains when extracted with A) ethanol, and B) hexane.

**A. Ethanolic Extraction of 10 Different Strains of *C. Sativa***			
Strain	Total phytocannabinoid (%)	Total terpene (%)	Terpenoid to phytocannabinoid ratio
Cs11_ct-I_	90.3	8.2	9
Cs12_ct-I_	94.4	5.6	6
Cs13_ct-I_	91.7	6.0	7
Cs14_ct-I_	85.8	9.7	11
SCBD_ct-III_	91.2	8.0	9
Arbel_ct-III_	92.5	7.5	8
Paris_ct-II_	90.6	9.4	10
71-P01_ct-III_	83.2	9.7	12
71-P38_ct-III_	96.4	5.6	6
61-P31_ct-I_	91.0	9.0	10
			**average**
			9 (± 2.3)
**B. Hexanoic Extraction of 5 Different Strains of *C. Sativa***			
Strain	Total phytocannabinoid (%)	Total terpene (%)	Terpenoid to phytocannabinoid ratio
Cs12_ct-I_	81.1	18. 1	22
38-P25_ct-II_	74.4	10.6	14
40-P38_ct-I_	81.1	16.4	20
61-P17_ct-I_	63.9	13.5	21
71-P21_ct-III_	72.3	16. 8	23
			**average**
			20 (± 3.5)

ct-I = chemotype I (high THC); ct-II = chemotype II (THC ≈ CBD); ct-III = chemotype III (high CBD).

## Supplementary Materials

Supplementary Figure S1: PCA correlations. PCA showed phytocannabinoid-terpenoid correlation. Only four terpenoids showed absolute and unique correlation with CBD: α-farnesene, α-famesene, guaiol and γ-eudesmol. The negative correlation of these terpenoids to THC shows that these compounds were not produced by any ‘high THC’ chemotype I plants. For THC, the variability of accompanying terpenoids and terpenoids is broader, containing over 17 different compounds. Supplementary Figure S2: Dose-effect curves of THC and CBD on the viability of MDA-MB-231 and HCT-116 cell lines. Data points were connected by non-linear regression lines of the sigmoidal dose-response relation. GraphPad Prism was used to produce dose-response curve and IC50 doses for THC and CBD. Supplementary Table S1: Dataset used for Principle Component Analysis (PCA) for the statistical calculation of correlation between terpenoids and phytocannabinoids. Relative amounts (%) of cannabinoids and terpenoids and terpenes extrapolated from the ethanolic extracts of the different strains, analyzed by GC/MS (for terpenoids) and HPLC (for phytocannabinoids). Supplementary Table S2: Statistical correlation between terpenoids and phytocannabinoids in the different strains analyzed. Supplementary Table S3: Summary of the effective cell death response based on cannabinoids-terpenoids correlation ratios/ranges.
