# Supplementary material for: Terpenoids and Phytocannabinoids Co-Produced in Cannabis Sativa Strains Show Specific Interaction for Cell Cytotoxic Activity
Source: Molecules. 2019 Aug 21;24(17):3031. doi: 10.3390/molecules24173031 (PMC6749504; doi:10.3390/molecules24173031)
Supplement: Supplementary file 1 [file molecules-24-03031-s001.pdf]

Supplementary Table 1. Dataset used for Principle Component Analysis (PCA) for the statistical calculation of correlation between terpenoids and phytocannabinoids. Relative amounts (%) of cannabinoids and terpenoids and terpenes extrapolated from the ethanolic extracts of the different strains, analyzed by GC/MS (for terpenoids) and HPLC (for phytocannabinoids).

	ARBEL	DQ	PARIS	SCBD	ROMA	CS11	CS12	CS13	CS14	38P25	38P28	40P38	61-P17	71P01	71P21	71P38	61P31
a-Pinene	33.70935	2.644466	1.741851	1.319345	2.644466	0.893542	0.264483	1.015493	0.09953	0	0	0.385925	0	0	0	0.34934	0.44263
Camphen	0	0.911742	0.253615	0	0.911742	0	0	0	0	0	0	0	0	0	0	0	0
b-Pinene	5.887975	4.105363	0.35797	0.11743	4.105363	0.404154	0.418617	0.284632	0.184364	0	0	0.424498	0	0	0	0.159257	0.250486
b-Myrcen	10.22105	8.291656	0.431333	0	8.291656	3.287882	1.513924	0.564683	0.684408	0	0	0.219599	0	0.448022	0	0.998748	4.697048
D-Limone	4.57028	19.5361	0	0	19.5361	0.205292	0.946712	0.63387	0.50752	0	0	0.64215	0	0	0.981158	0.117499	0.630003
b-Ocimen	0	0	0	0	0	0.098133	1.646752	0.537605	0.878997	0	0	0.355473	0	0.218558	0.630312	0.138345	0
Linalool	0.891147	2.01912	0	0	2.01912	0.24625	5.884169	0	0	0	0	0.164867	0	0	0	0.059334	0.15506
Fenchol	0.929556	2.333249	0	0	2.333249	0.023359	0	0.135476	0.099751	0	0	0	0	0	0	0.0401	0.069953
Terpinole	0.722785	0	0	0	0	0	0	0	1.943966	0	0	0	0	0	0	0	0
Thymol	0	0	0	0	0	0	0.109916	0	0.08119	0	0	0	0	0	0	0	0
trans-2-pi	0	0	0	0	0	0.078786	0	0.090596	0	0	0	0	0	0	0	0.029636	0.030157
Borneol	0.514094	0.484349	0	0	0.484349	0.03821	0.207334	0	0.082301	0	0	0	0	0	0	0.106664	0.077255
a-Terpine	0.7903	1.229534	0	0	1.229534	0.062221	0.12897	0.129299	0.117268	0	0	0	0	0	0.947233	0.071213	0.039442
b-Caryoph	8.160237	22.42164	1.506981	0.442656	22.42164	0.315696	2.015453	1.529001	1.71554	1.579108	1.092053	0.557975	2.296565	0.261311	3.653075	0.714533	1.511396
trans-a-Be	1.029883	0	0.473055	0.300809	0	0	0.494297	0	0.345588	1.617721	0.399074	0.282772	0.886647	0.149725	0	0.028851	0.036501
a-Elemen	0	1.724134	0	0	1.724134	0	0	0	0	0	0	0	0	0	0	0	0
c-Elemene	0	0	0	0	0	0.126301	0	0	0	0	2.534294	0	0	0	0	0	0
a- Guaien	0	0	0	0	0	0	0.336772	0	0.223787	0	1.353014	0	0	0	0	0	0
b-Famese	0	0	0	0	0	0	0.760676	0.025688	0.593275	0.559401	0.906853	0	0	0	0	0	0
Humulene	2.436843	5.797492	0.292432	0.163032	5.797492	0.079284	0.626788	0.625335	0.512221	0.54166	0.544391	1.154307	0.978989	1.65248	1.075531	0.255499	0.030362
a-Bisabole	1.647701	0	0	0	0	0	0.101266	0	0	0	0	0	0.368977	0	0	0	0
Alloaroma	0	1.286662	0		1.286662	0	0	0	0	0	0	0	0	0	0	0.022943	0.072463
d-Selinen	0	0	0.259246	0.231125	0		0.292621	0.30291	0.191061	0	0	0	0	0	0	0.022964	0.077007
a-Farnese	0	0	0	0	0	0.024047	0	0.150563		0	0	0	0	0	0.290514	0.090038	0.19709
Patcholen	6.549357	0	0	0	0	0	0	0	0	0	0	0	0	0	0	0	0
a-Bisabole	0	1.457027	0.464192	0.418879	1.457027	0.03195	0	0	0	0	0	0	0	0	0	0	0
a-selinene	5.568251	0	0	0	0	0	0.220107	0	0.190713	0	0	0	0	0	0	0	0
a-Famese	0	0	0	0	0	0	0.265049	0	0.202643	0	0	0.192477	0	0.221042	0	0	0
cis-a-Buln	0	0	0	0	0	0	0.598167	0	0.231727	0	0	0	0	0	0	0	0
a-Muurole	0	1.179635	0	0	1.179635	0	0	0	0	0	0	0	0	0	0	0	0
b-Cadiner	0	1.557537	0	0	1.557537	0	0	0	0	0	0	0	0	0	0	0	0
Valencene	0	4.792179	0	0	4.792179	0	0	0	0	0	0	0	0	0	0	0	0
p-Panasin	0	0	0	0	0	0.09554	0	0	0	0	0	0	0	0	0	0	0
b-sesquip	0	0	0	0	0	0	0.149214	0	0.113737	0	0	0	0	0	0	0	0
Selina-3,7	0	5.670836	0	0	5.670836	0.062702	0	0	0	0	0	0	0	0	0	0	0
cis-a-Bisal	0		0	0	0	0	0.120748	0	0.125088	0	0	0	0	0	0.156651	0	0
c-Elemene	0	0.867778	0	0	0.867778	0.088556	0	0	0	0	0	0	0	0	0	0	0
Nerolidol	0	0	0	0	0	0.225839	0.103109	0	0.179499	0	0	0	0	0	0	0	0.028811
Guaiol	2.620685	0	0.841278	0.35382	0	0.544872	0	0	0	2.233998	5.081673	3.872181	6.063161	1.599367	3.901939	0.67002	0.133819
c-Eudesm	2.833333	0	0.772596	0.384682	0	0.054038	0	0	0	2.125368	5.106661	4.845272	1.525972	2.29092	2.375696	0.361172	0.16158
a-Eudesm	2.34162	0	0.142822	0.525203	0	0.701159	0	0	0	0	0	0	0	0	0	0.304095	0.108236
Bulnesene	0	0	0	0	0	0.26109	0	0	0	0	0	0	0	0	0	0	0
Guaienol	0.869977	0	0	0	0	0	0	0	0	0	0	0	0	0	0	0	0
a-Bisbolol	2.007734	0	86.0225	0.315687	0	0.110899	0	0	0	0.344756	4.094447	3.277259	0.448446	2.739637	2.345779	0.401166	0.178486
neophitac	0	0	0	0	0	0	0	0	0	0.641296	0.791535	1.879748	0	0.144882	0.439691	0	0
Phytol	0	0	0	0	0	0.031707	0	0	0.168044	2.307631	0.924184	0	1.928424	0.774587	3.887693	0	0
CBL	0	0	0	0	0	0.062869	0	0	0.037972	0	0	0	0	0	0	0	0
CBD	5.69784	0	3.590705	80.82148	0	2.538372	0.40273	0.608904	2.272786	29.10697	23.62056	75.43102	57.18808	78.98588	67.7221	89.67826	46.02702
CBC	0	0	0	0	0	1.922402	1.719031	0.435065	0.279471	3.898327	3.498281	2.751087	0.840887	1.009289	0.852576	1.454077	0.764841
d8-THC	0	0	0	0	0	0	2.815154	0	0.070191	0	0	0.423982	0	0	0	0	0
THC	0	9.666126	1.823267	3.050723	9.666126	81.86516	83.63616	69.90952	76.21782	41.37048	31.90565	2.515617	5.588576	2.699287	3.277304	3.173532	42.9347
CBG	0	0	0	9.742831	0	2.383828	4.686823	3.428362	5.414096	0	0	0	0.322073	0.487709	0.432878	1.479963	0.873183
CBN	0	0	0	0	0	0.477259	0.453295	0.259963	0.518045	0	0	0	0	0	0	0.244282	0.445934

Supplementary Table 2. Statistical correlation between terpenoids and phytocannabinoids in the different strains analyzed.

A. Strong Correlation			B. Non-Specific Correlation (Independent)			C. Relative Correlation		
Metabolite	CBD	THC	Metabolite	CBD	THC	Metabolite	CBD	THC
β-Ocimene	-0.19	0.57	α -Pinene	-0.25	-0.24	α -Farnesene	0.25	0.03
Linalool	-0.42	0.32	Camphene	-0.40	-0.25	α -Famesene	0.06	0.29
Terpinolene	-0.29	0.30	β -Pinene	-0.45	-0.29	CBC	0.17	0.23
Thymol	-0.34	0.62	β-Myrcene	-0.47	-0.14	CBG	0.07	0.37
trans-2-pinanol	-0.21	0.54	D-Limonene	-0.40	-0.23			
γ-Elemene	-0.08	0.06	Fenchol	-0.43	-0.25			
α - Guaiene	-0.17	0.21	Borneol	-0.48	-0.16			
β-Famesene	-0.31	0.52	α -Terpineol	-0.32	-0.30			
δ-Selinene	-0.30	0.44	β -Caryophyllene	-0.42	-0.26			
cis-a-Bulnesene	-0.32	0.58	trans- α -Bergamontene	-0.09	-0.04			
p-Panasinsene	-0.23	0.44	α -Elemene	-0.36	-0.21			
β-sesquiphellandrene	-0.34	0.62	Humulene	-0.32	-0.34			
cis- α -Bisabolene	-0.08	0.33	α -Bisabolene	-0.18	-0.23			
Nerolidol	-0.40	0.76	Alloaromadendrene	-0.35	-0.21			
Bulnesene	-0.23	0.44	Patcholene	-0.20	-0.22			
CBL	-0.32	0.60	α -Bisabolene	-0.34	-0.29			
δ^8-THC	-0.20	0.43	α -selinene	-0.22	-0.19			
CBN	-0.30	0.83	α -Muurolene	-0.36	-0.21			
exo-THC	-0.24	0.45	β -Cadinene	-0.36	-0.21			
Guaiol	0.37	-0.40	Valencene	-0.36	-0.21			
γ-Eudesmol	0.33	-0.38	Selina-3,7-diene	-0.36	-0.21			
neophitadiene	0.33	-0.20	γ-Elemene	-0.38	-0.18			
Phytol	0.31	-0.20	α -Eudesmol	-0.14	-0.17			
			Guaienol	-0.20	-0.22			
			α -Bisbolol	-0.20	-0.23			

Supplementary Table 3. Summary of the effective cell death response based on cannabinoids-terpenoids correlation ratios/ranges.

Cannabinoid	IC50 (μg/ml)	Terpenoids	Terpenoids Range (μg/ml)	Range of Cell Death Range (%)	Synergistic Cann:Terp Ratio	Maximum Synergistic Cell Death (%)
			A. *MDA-MB-231 cell line*			
THC	10	β -Thujene<br>α -Pinene<br>Camphene<br>β -Pinene<br>α -Phellandrene<br>α -Terpinene<br>β -Phellandrene	1-2	30	10:1	40
CBD	10	α -Guaiene<br>γ-Selinene<br>Guaiadiene<br>δ-Selinene<br>Selinadiene<br>β -Maaliene	3-0.5	39	10:2	80
			B. *HCT-116 cell line*			
THC	20	β -Thujene<br>α -Pinene<br>Camphene<br>β -Pinene<br>α -Phellandrene<br>α -Terpinene<br>β -Phellandrene	0.5	20	10:0.25	20
CBD	20	α -Guaiene<br>γ-Selinene<br>Guaiadiene<br>δ-Selinene<br>Selinadiene<br>β -Maaliene	2-0.5	33	10:0.25	50

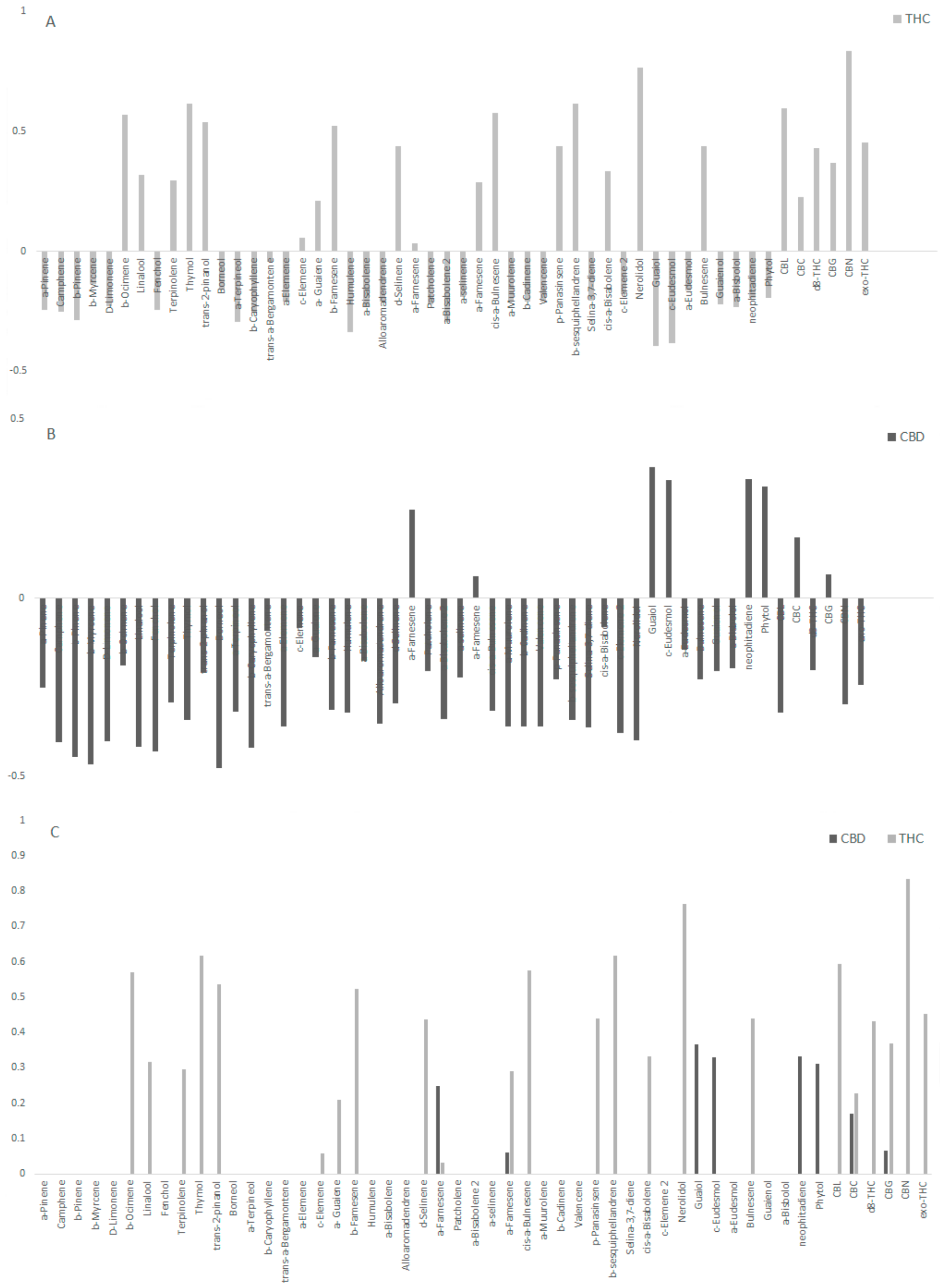

Supplementary Figure 1. PCA correlations. PCA showed phytocannabinoid-terpenoid correlation. Only four terpenoids showed absolute and unique correlation with CBD: α-farnesene, α-famesene, guaiol and γ-eudesmol. The negative correlation of these terpenoids to THC shows that these compounds were not produced by any ‘high THC’ chemotype I plants. For THC, the variability of accompanying terpenoids and terpenoids is broader, containing over 17 different compounds.

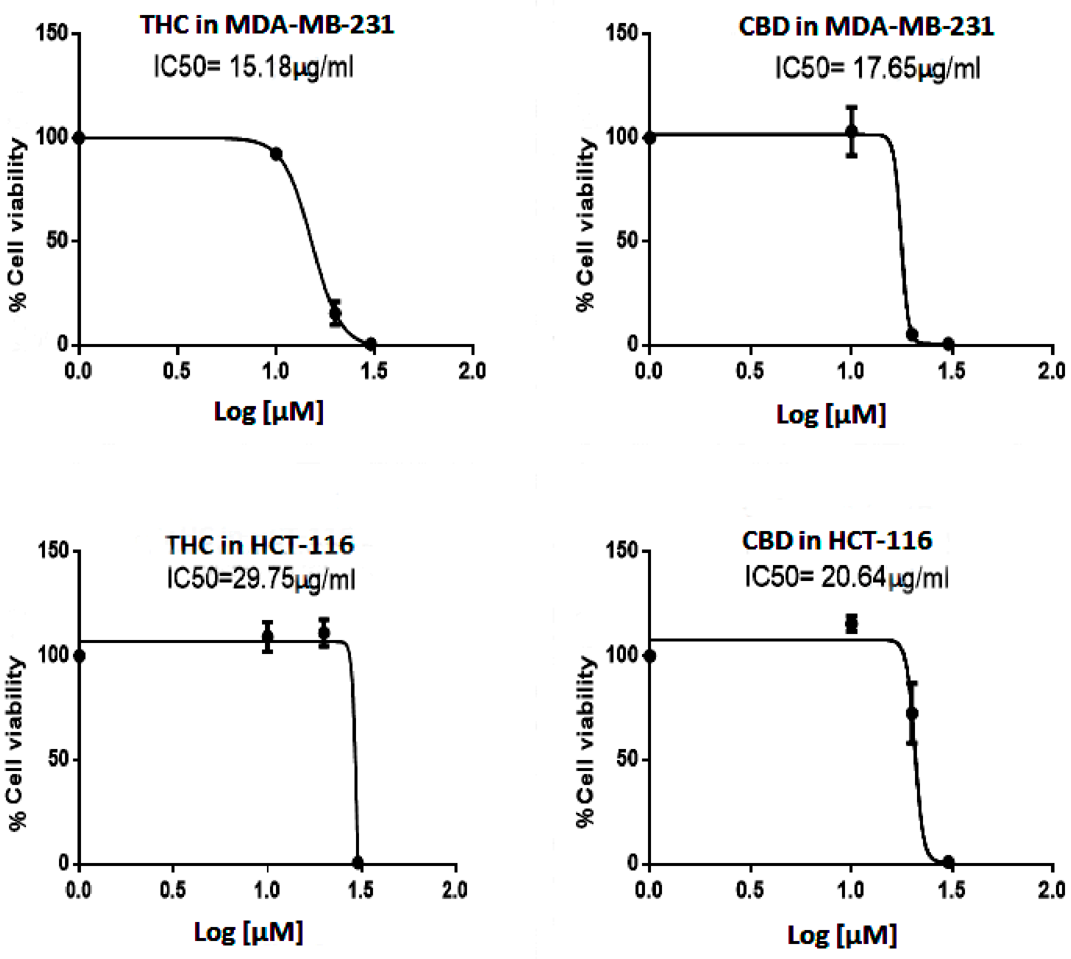

Supplementary Figure 2. Dose-effect curves of THC and CBD on the viability of MDA-MB-231 and HCT-116 cell lines. Data points were connected by non-linear regression line of the sigmoidal dose-response relation. GraphPad Prism was used to produce dose-response curve and IC50 doses for THC and CBD.
